# Draft Genome Sequence of *Paenibacillus amylolyticus* Heshi-A3, Isolated from Fermented Rice Bran in a Japanese Fermented Seafood Dish

**DOI:** 10.1128/genomeA.00218-16

**Published:** 2016-03-31

**Authors:** Sayuri Akuzawa, Junko Nagaoka, Motoki Kanekatsu, Eri Kubota, Rumi Ohtake, Tomonori Suzuki, Yu Kanesaki

**Affiliations:** a Department of Nutritional Science and Food Safety, Tokyo University of Agriculture, Tokyo, Japan; b Department of Biological Production, Tokyo University of Agriculture and Technology, Tokyo, Japan; c NODAI Genome Research Center, Tokyo University of Agriculture, Tokyo, Japan

## Abstract

*Paenibacillus amylolyticus* strain Heshi-A3 was isolated in Fukui prefecture, Japan, from fermented rice bran in Heshiko, a traditional dish that is produced by aging salted mackerel with fresh rice bran at an ambient temperature for around 7 months to over one year. Here, we report the draft genome sequence of *Paenibacillus amylolyticus* strain Heshi-A3.

## GENOME ANNOUNCEMENT

Heshiko, a Nukazuke product categorized as fermented seafood in Japan, is produced with fresh rice bran, using only a carbohydrate source during fermentation. After the fermentation period, fresh rice bran changes from a powder into a paste with a strong umami and sour taste. Some studies on fermented seafood have identified the dominant bacterial microflora present during the processing of Heshiko (1), the changing chemical components, and the relationship between sensory evaluation and free amino acids with peptides (2). Previously, we isolated the bacterium *Oceanobacillus picturae* Heshi-B3 from fermented rice bran in Heshiko and analyzed its draft genome sequence (3). However, still only a few reports have analyzed the genome sequences of bacteria isolated from fermented rice bran in Heshiko. Here, we report the draft genome sequence of a newly isolated bacterium from Heshiko belonging to the genus *Paenibacillus*. According to the similarity of genetic sequences, we named this strain *Paenibacillus amylolyticus* Heshi-A3.

Bacterial cells were cultured in medium (1% soluble starch, 0.5% yeast extract, 0.5% peptone, 0.1% K_2_HPO_4_, 0.02% MgSO_4_·7H_2_O, and 5% NaCl) at 30°C and were collected by centrifugation (10,000 × *g*, 15 min, 4°C). The cell pellet was treated with cell lysis solution (20 mg/ml lysozyme, 20 mM Tris-HCl [pH 8.0], 2 mM EDTA, 1.2% Triton X-100) for 30 min at 37°C. The bacterial genomic DNA was prepared using the DNeasy blood and tissue kit (Qiagen, Valencia, CA, USA). Five micrograms of genomic DNA dissolved in Tris-EDTA buffer was digested by a Covaris S-2 sonicator (Covaris, Woburn, MA, USA) with an average size of 500 bases. Construction of a DNA library and sequencing by a massively parallel sequencer (MiSeq; Illumina KK, Tokyo, Japan) was shown in our previous study (3). The sequenced reads were screened by a quality score higher than the Phred score 30 and were each trimmed 10 bases from both the 5′ and 3′ ends. The trimmed reads were assembled *de novo* by CLC Genomics Workbench version 7.5 (Qiagen, Valencia, CA, USA). Among the assembled contigs, several that were derived from minor contaminated microorganisms were removed by a BLAST search. Finally, the 15 contigs (>1 kb) were assembled with a total length of 6,760,550 bp, the longest contig being more than 4 Mb. Annotation of the contigs was performed by MiGAP (4). As a result, 12 rRNAs, 93 tRNAs, and 5,968 coding sequences were identified. Our sequencing data will largely contribute to future studies to understand the genetic variation among the species of *P. amylolyticus*.

### Nucleotide sequence accession numbers.

The whole-genome shotgun project of *Paenibacillus amylolyticus* Heshi-A3 has been deposited in DDBJ/ENA/GenBank under the accession number BCNV00000000. Sequences and annotations of the contigs are available under the accession numbers BCNV01000001 to BCNV01000015.
